# Cultural aging stereotypes in European Countries: Are they a risk to Active Aging?

**DOI:** 10.1371/journal.pone.0232340

**Published:** 2020-05-15

**Authors:** Rocío Fernández-Ballesteros, Ricardo Olmos, Lourdes Pérez-Ortiz, Macarena Sánchez-Izquierdo

**Affiliations:** 1 Dept of Psychobiology and Health, Autonomous University of Madrid, Madrid, Spain; 2 Dept of Methodology, Autonomous University of Madrid, Madrid, Spain; 3 Dept of Sociology, Autonomous University of Madrid, Madrid, Spain; 4 Dept of Psychology, Universidad Pontificia Comillas, Madrid, Spain; Saint Peter's University, UNITED STATES

## Abstract

A growing body of literature acknowledges the association between negative stereotypes and individual components of active aging, but very few studies have tested this association, at both individual and population levels. The Stereotypes Content Model (SCM) states that the cultural aging stereotyping of higher warmth than competence (called paternalistic or ambivalent prejudice) is universal. Our aims in this study are to test the extent to which the universality of this stereotype is confirmed in European Countries as well as how far "positive", "negative" or "ambivalent" views towards older people, and other negative attitudes such as prejudice and behaviours such as discrimination, predict active aging assessed both at individual and population levels. We have analyzed data from the European Social Survey-2008 (ESS-2008), containing SCM stereotypical and other appraisal items (such as direct prejudice and perceived discrimination) about adults aged over-70 from 29 European countries. First, SCM cultural stereotypes about older adults (“friendly”, “competent”, and “ambivalent”) were calculated; secondly, after developing a typology of countries based on their “negative”, “ambivalent” and “positive” views about older adults, the universality of cultural stereotypes was tested; thirdly, taking into consideration ESS data of those older persons (over 70s) who self-reported indicators of active aging (health, happiness, satisfaction and social participation), multilevel analyses were performed, taking our inter-individual measure of active aging as dependent variable and our stereotypical classification (positive/negative/ambivalent), direct prejudice and perceived discrimination as predictors; finally, relationships between stereotypical and appraisal items on older adults were examined at population level with country data from Active Aging Indexes. Our results show cultural stereotypes about older people (more friendly than competent) are widespread in most European countries, and negative cultural views of older adults are negatively associated with active aging both at individual and population level, supporting that negative cultural views of older adults could be considered as a threat to active aging.

## Introduction

It is common knowledge that stereotypes are exaggerated views or conceptions about members of a group based on simplistic generalizations without considering their individual characteristics. As Hummert [1] pointed out, aging stereotypes are cognitive dynamic structures, engender negative attitudes or evaluative responses and influence behaviours against both the aging group and older adults. Moreover, age stereotypes can refer to a given culture (cultural stereotypes), to a person (personal stereotypes) or can even refer to one’s own individual (self-stereotype) or self-perception of aging. This cognitive semantic network is called “ageism” by Butler [2] (in the same way as sexism or racism), formed by these three connected elements of stereotyping, prejudice, and discrimination against people on the basis of their age, being defined as “unfair treatment of people who are becoming old or who are old” (https://dictionary.cambridge.org/ageism), which is complemented with its emotional consequences or prejudices against group members and, finally can provoke behavioural discrimination against a certain person or group.

Since this study seeks to investigate whether this stereotypical network is affecting older adults’ active and healthy life, let us first examine briefly both the diversity of these stereotypical concepts and the strong association between stereotypes and health.

### Stereotypical concepts

Going back to our key concept of stereotypes, although the specialized empirical literature consistently reveals negative stereotypical views about aging and older adults, we must briefly describe the set of stereotype concepts and the methodology behind them: cultural, personal, self-stereotypes, or even self-perception of aging (a very close attribute; for a review see [1]). These concepts are investigated with a diversity of methodologies, supported by experimental, implicit or even neurophysiological techniques. They are usually measured through verbal reports administering positive and/or negative verbal items, and also different types of response scales.

Clearly, this is not the place to review these methodological approaches. In this study we must highlight an important aspect: who is holding the view about aging: whether this refers to the individual/s who report their view–in the first person–or whether it is the culture or context to which the informant belongs to whom that vision or conceptualization is attributed, thereby distinguishing between personal, group or cultural stereotypes [3].

Fiske and her group, from the Stereotype Content Model theory (SCM) [4, 5], maintain that it is the culture to which both the individual respondent and the group that holds the stereotype belong, as formulated in the question: *"*How likely is it that most people, in your country view those……*"*. From this perspective, authors avoid positive/negative valence postulating two stereotypical dimensions, both positive: warm/friendly and competent/intelligent; these are applicable to various social groups (young/old, men/women, etc.), and depending on their differential score (high/low), they are distributed across four quadrants, with the elderly in the first quadrant (higher warmth, lower competence). These authors consider that "some groups, including elderly people, face a more complex brand of prejudice that comprises both negative and subjectively positive beliefs, feelings, and behaviours" [5, 6, 7] and this study is based on the SCM.

Older people, according to the SCM, are thus perceived by a given culture/country as an identifiable group with assigned stereotypes: kind, thankful, or warm, but not very competent, intelligent or efficient. This prototypical response profile focusing on attributes of kindness and competence, labelled by the authors as "paternalistic prejudice", is considered to be an "ambivalent" outlook or stereotype which describes elderly people as a harmless group which neither threatens the community nor has the capacity to do so. Furthermore, they need others (experts, etc.) to take responsibility for them who express feelings of pity and sympathy towards the older adults.

In addition to the cultural nature of the stereotype stemming from SCM, it is important to highlight two relevant aspects:

1. In terms of methodology, an assessment tool, the SCMQ (*Stereotypes Content Model Questionnaire* [5]) has been developed, consisting of 10 items, 5 each for the “warmth" and "competence" dimensions (with a scale of 0–5), and which has good psychometric properties with high internal consistency. However, when these two stereotypical dimensions from the SCM are introduced in international population surveys (as in the European Social Survey-ESS or the World Value Study-WVS), only two items, "friendly" and "competent", both positive, are used to represent the two dimensions, thereby reducing the potential for psychometric research.
2. From an empirical point of view, the SCM is based on correlational or experimental studies with intentional samples, university students from the USA and some other countries. Therefore, making results generalisable is difficult. The stereotype content model has received considerable empirical support (for a review see [8]). Cuddy et al. [9] examined the universality of the stereotype content model across individualist European countries (Belgium, France, Germany, The Netherlands, Portugal, Spain, and the UK) and three East Asian collectivist countries (Hong Kong, Japan, and South Korea), and results confirmed that older people are categorized in the ambivalent stereotype category as warm but not very competent.

The authors claim universality for the cultural stereotype of the elderly, and the results of our own research uphold the existence of widespread stereotypical views of the elderly as more warm than competent [10, 11]. Following this line, our research program into aging stereotypes and older persons draws on different theoretical models [5, 12, 13] and has been implemented in different populations (Mexico, Germany and Spain [14]). It has involved large representative samples of older people and professionals working with older people, from healthy seniors enrolled on university programs or in senior centres to elderly people with functional difficulties attending day care centres or living in assisted facilities. Hence the study by Abrams, Russell, Vauclair, and Swift [15,16], using data from the European Social Survey (ESS) 2008, showed that people aged over 70 were least likely to be attributed competence; furthermore, age discrimination was the most widely experienced form of discrimination across Europe for every age group.

In sum, the objective of this study is to test the universality of cultural stereotypes in representative samples across European countries.

### Stereotypes and health

Aging stereotypes, prejudice and discrimination, in short ageism, appear to influence physical and mental health, well-being and, therefore healthy and active aging (see [17]).

A considerable amount of research on the effects of negative stereotypes of aging has focused on stereotype threat, which refers to the fear that arises in situations in which a member of the group is at risk of conforming to a negative stereotype about their age group associated with poor performance in a given domain, mainly in those related with cognitive or physical performance [18]. Thus, stereotype threat involves the explicit activation of stereotypes, mediated by anxiety and fear, conforming to the cultural stereotype [1, 19, 20]. Hence the study by Abrams et al. [15], based on European Social Survey (ESS) 2008 data, showed the belief among the younger respondents (aged 15–24) that those over 70 present a greater economic burden, a burden on health and public services and thus an economic threat; in addition, respondents approaching retirement age and those who are likely to be retired (aged over 65) were more worried about employers favouring younger people.

Stereotype threat can happen also in health care situations, as Burgess et al. [21] pointed out, warning physicians and health providers: “patients’ experience of stereotype threat in clinical settings may be one contributor to health care disparities. Stereotype threat occurs when cues in the environment make negative stereotypes associated with an individual’s group status salient, triggering physiological and psychological processes that have detrimental consequences for behavior” (p. 169). In this situation, the older adult can be assigned to a given medical category, including a disease, a given risk factor, an unhealthy lifestyle and/or reduced competence simply because of their age. For example, Abdou and Wheathon [22] examined the stereotype threat situation in a Health and Retirement Study (HRS; in a random selection of *N* = 1,479 individuals). Logistic regression was used to test whether healthcare stereotype threat was associated with self-rated health, reported hypertension, and depressive symptoms, as well as with healthcare-related outcomes, including physician distrust, dissatisfaction with healthcare, and preventive care use. Results showed that 70% reported healthcare stereotype threat with respect to one or more aspects of their personal data. Moreover, the authors reported healthcare stereotype threat correlated with higher physician discontent with health care, poorer mental and physical health (i.e., self-rated health, hypertension, and depressive symptoms), and lower probability of receiving the influenza vaccine.

From a similar perspective Cuddy and Fiske [23], in their seminal article *Doddering but Dear*, stated: “…elderly people are stereotyped as incompetent but also warm. People view them ambivalently as physically and cognitively inept but socially sensitive….(therefore)…from workplaces to medical settings, stereotyping of elderly people manifests itself throughout discriminatory communication and treatment” (pp 17–18). Furthermore, Durante et al. [24] showed that more unequal societies (Gini index) report more ambivalent stereotypes. We found similar results in social care contexts when we tried to examine the role of the competence and warmth stereotypes of older adults held by professional caregivers from the stereotype content model (SCM) perspective [11]. Our results showed the influence of caregivers’ cultural stereotypes on the type of care, as well as on their professional behaviours and on older adult functioning. In sum, caregivers’ cultural stereotypes could be considered as a central issue in older adult care, since they mediate the triangle of care: caregivers/older adults/type of care; therefore, much more attention should be paid to this psychosocial care component.

Another perspective is taken by Levy [25] (see also [26]); she posited a new theoretical psychosocial theory with two main components: age stereotypes are internalized (unconsciously embodied) across the life span and transformed into self-stereotypes or self-perception of aging which then affect health outcomes. Several studies have confirmed the relationship between individual self-perception of aging and health by influencing health behaviours such as physical activity (e.g. [27, 28]), preventive health behaviours (low alcohol consumption, regular doctor visits, eating a proper diet, maintaining a healthy weight, using a car seatbelt, exercise, avoiding tobacco and medication compliance [4], and the tendency of older adults to refuse life-prolonging interventions, weakening elderly people's will to live [29].

Furthermore, age stereotypes not only affect older persons’ behaviours but also have significant long-term effects on health outcomes. Levy et al. [25] analyzed data from a subset of participants in the Ohio Longitudinal Study of Aging and retirement (OLSAR). Their results showed that having more positive aging self-perceptions predicted self-reported functional health and longevity. Moreover, several studies have found evidence of a deterioration in postural stability and balance [30], and older adults are more likely to suffer from cardiovascular disease [31] and to experience hospitalization [32], dependency [33], respiratory mortality [34]. Moreover a recent study based on BLSA [35] showed participants with more negative age stereotypes earlier in life having elevated counts of biomarkers related to Alzheimer’s disease in a post-mortem brain autopsy.

In sum, when the stereotype threat model was reviewed, we concluded that both in health and social care contexts, the effect of cultural stereotypes on older adult health, mediated by these health or social professional or caregiver behaviours, can be identified. Following Levy´s embodiment theory, age stereotypes affect health through very complex unconscious mechanisms which transform cultural age stereotypes into self-stereotypes influencing individual behaviours and affecting health and even longevity.

Let us now turn from health to another broader concept: active aging. In 2002, on the occasion of the General Assembly of Aging approving the Madrid Second International Plan of Action on Aging-MIPA [36], the World Health Organization [37] enlarged the concept of healthy aging, coining the term active aging as “the process of optimizing opportunities for health, participation and security in order to enhance the quality of life as people age"(p.12). In the same document, WHO emphasized the negative effect of stereotypes through the development of this process.

We partially tested the relationships between active aging and views about age and older adults from two perspectives: 1) the effect of perceived discrimination in active aging: using a structural equation modelling framework on a sample from three countries (Germany, Spain and Mexico), we showed that perceived discrimination (a consequence of stereotypes) negatively predicts active aging [17]. 2) we have dedicated several research projects highlighting the beneficial effects of promoting active aging programs to reduce cultural stereotypes and improve self-perception of aging [38].

Recently, Swift, Abrams, Lamont and Drury [39] criticised the scarce empirical research on the impact of stereotypes, attitudes, and discrimination toward age, called “ageism” [2] (see also: [40, 41]), which can affect active aging. Moreover, they proposed a micro-theory through the “Risks of Ageism Model (RAM)" positing hypotheses about how ageism could affect active aging (health, participation and security) through a set of their determinants (socio-economic, health and social services, behavioural, personal, etc.), mediated both by the mechanism of aging (stereotype embodiment and stereotype threat, the ageism target). Although we consider that “ageism” cannot be reduced only to these psychosocial components, we agree with the hypothesis that public policies–promoting active aging and related concepts–should pay greater attention to aging stereotypes. In summary, according to the available empirical data regarding aging stereotypes and healthy and active aging, it can hypothesized that stereotypes about older adults can have consequences for healthy and active aging.

In conclusion, focusing on the cultural stereotypes approach developed by the SCM, this study aims to ascertain to what extent the stereotypical high-warmth, low-competence pattern culturally held regarding older adults is universal, and whether stereotypes have an effect on both population and individual active aging.

The ESS-2008, which includes useful items referring to stereotypes, prejudices and discrimination about older people, is used (for a review: [42, 43]). This data source offers a unique opportunity in representative population samples from 29 countries to investigate and test our hypotheses: 1) whether cultural aging stereotypes of high warmth and low competence are attributed to older individuals as predicted by the SCM, and 2) whether the stereotype network is associated with both individual and population variables related to or predicting active aging.

## Materials and methods

### Ethical considerations

Our study uses a database, SSE-round 4, in which the personal data and information is encoded and anonymised. Furthermore, the European Social Survey specifies that: 1) In accordance with the ESS ERIC Statutes (Article 23.3), the ESS ERIC subscribes to the Declaration on Professional Ethics of the International Statistical Institute. 2) The Research Ethics Committee reviews applications for studies for which the ESS ERIC is directly responsible, that is, which it directly contracts.

### Participants

The sample used was extracted from the European Social Survey-ESS (round 4, 2008), comprising a total of 54,545 participants from 29 countries, 54.4% women, with an average age of 47.4 years (*SD* = 18.38). Potential sex and age differences in the different samples were examined, revealing slight differences in the proportion of women per country (F(28,54492) = 13.76, *p* < .01, *η*2 = .007), with Ukraine the country with the highest proportion of women surveyed (62.6%) and Germany having the smallest (47.3%). There are also some age differences between the countries (F(28,54340) = 35.51, *p* < .01, *η*2 = .018), with Turkey the country whose average age was lowest (39.75) and Portugal having the highest average age (52.35).

The means and SDs of the cultural vision of "friendly" and "competent" were taken from the country sample individuals, and the friendly-competent difference, so-called "paternalistic or ambivalent prejudice" and the self-reported evaluative items “direct prejudice” and “perceived discrimination”, theoretically considered as the consequence of stereotypes, were also included. Finally, the total mean was calculated for the 29 countries in all variables.

Given that the group to which this stereotypical cultural attribution applies comprises people aged over 70, and in order to operationalize active aging at an individual level, following WHO definition (satisfaction, happiness, health, participation/social network), the sample of those over 70 was selected from the total sample, comprising 7,600 participants (59.2% women, average age, 76.75, *SD* = 5.37).

Finally, let us emphasize once more that our results referring to country cultural stereotypes and related concept are taken from the ESS total country samples, which is also the case when we examine active aging at the population level through the Active Aging Index (AAI) (by country). Finally, when examining active aging at individual level, we used data from a sample of adults over 70.

### Assessing cultural images and other related concepts

Two types of items referring to people over 70 are included in the ESS: cultural stereotypes of warmth and competence. Cultural stereotypes are examined through the meta-perception of the ESS respondents, that is, the response to *"*How are people over 70 perceived in your country?" with the following format: "tell me how likely it is that most people in [country] view those over 70s. . . as friendly and. . . as competent "; the answers are collected using a Likert-type scale ranging from 0 (Not at all likely) to 4 (Very likely). As pointed out above, these two items represent a reduction of the SCM Questionnaire, composed, as indicated in the Introduction, of 10 items (5 for each dimension). Unfortunately, this fact, among other difficulties, limits the comparability to the two-item ESS and prevents the estimation of certain metric properties as well as the comparability with other results from the original measures, but it does allow us to make comparisons made by the WVS using the same items and metrics. Nevertheless, we have tried to compensate for this by using a multiplist approach, examining multiple indicators and performing multiple analyses to test hypotheses [44].

In order to test our first hypothesis about the universality of the SCM [2, 45] cultural aging stereotype, it was necessary to calculate the difference (subtraction) between the scales "friendly" and "competent", which yielded an “ambivalent” stereotypical view (also called “paternalist prejudice”).

For our second hypothesis, that is, the extent to which cultural stereotypes predict active aging, we must take into account that, as has been mentioned in the Introduction, the SCM operationalizes cultural stereotypes based on the combination of two conditions, both positive: warmth and competence. Since the literature emphasizes the confrontation between positive and negative views (e.g. [4, 41]), in order to link stereotypes and health (therefore, active aging), it was decided to classify individuals into three groups: negative, positive and ambivalent view. The negative cultural vision group comprises those individuals below the mean in both variables (M = 2.91 in friendly and M = 2.45 in competent). The ambivalent cultural vision group includes those individuals who scored below the mean in the competent dimension (< 2.91) and above the mean in the friendly dimension. Those individuals above the mean in the two dimensions were assigned to the group called positive cultural, and, finally, a fourth group called “others” was created for those individuals who scored above the mean in the competent dimension, but below the mean in the friendly dimension. Consequently, we created four groups through both cultural stereotype variables, instead of just the difference between friendly and competent. Table 1 shows the percentages of the whole sample across the four groups.

**Table 1:** Number of individuals and percentages in the proposed four cultural views classification.

		In this Country most people view those over 70 as competent				
		0	1	2	3	4
In this Country most people view those over 70 as friendly	0	NEGATIVE VIEW (N = 11,603; 21.3%)			OTHERS * (*N* = 3,815; 7.0%)	
	1					
	2					
	3	AMBIVALENT VIEW (*N* = 15,467; 28.4%)			POSITIVE VIEW (*N* = 23,660; 41.7%)	
	4					

* OTHER, can be considered also “the Competent group” (that is, those individuals above the mean in competent ESS variable and below the mean in Friendly ESS variable) also took part of the reference group jointly with positive vision group in multilevel analysis (see later) because two reasons: there is a small percentage of people in this group (only a 7%) and because it did not contribute as a significant predictor of active aging and then was merged with positive (reference) group

Furthermore, out of the SCM proposed by Fiske and Cuddy, the ESS has two more items associated with stereotypical views which are theoretically considered as their outcomes; as we can read below, these items will be helpful in order to triangulate this classification; they are: 1) personal appraisal or "direct prejudice" by Abrams, Vauclair and Swift [46], referring to the respondent's personal valuation of E34 (card 55) of the ESS: *"*Tell me overall, how negative or positive you feel towards people over 70?*"* (Scale 0–10: 0 = Extremely negative, 10 = Extremely positive) and, 2) perceived discrimination is stated as: “how often, in the past year, anyone has shown prejudice against you or treated you unfairly because of age”, item E34 (card 56) (Scale 0 = Never to 5 = Very often).

### Active aging component operationalization and measurement

The World Health Organization defines active aging as "the process of optimizing opportunities for health, participation and security in order to enhance the quality of life as people age "([38], p.12). Since the ESS Questionnaire contains items related to criteria involved in Active Aging, it was operationalized through the following items about *health* (“How is your health in general” scaled as 1 = very good to 5 = very bad; *M* = 2.98, *SD* = 0.97), happiness (“How happy would you say you are” scaled as 0 = extremely unhappy to 10 = extremely happy; *M* = 6.54, *SD* = 2.36), *satisfaction (“how satisfied are you with your life as a whole nowadays*?” scaled as 0 = extremely dissatisfied to 10 extremely satisfied; *M* = 6.33, *SD* = 2.59), and participation (“About how many friends, other than members of your family, do you have who are aged over 70” scaled as 1 = None to 5 = 10 or more; *M* = 3.40, *SD* = 1.27).

In order to test our hypothesis regarding the extent to which cultural stereotypes are associated with active aging reported by adults over 70s, we calculated active aging as follows: using principal component analysis, we analysed whether one factor (component) accounted for the four variables (health, happiness, satisfaction and participation). The eigenvalue of the first factor was 2.227 (explaining 55.67% of the whole variance) and the three other eigenvalues < 1. The correlations (or factor loadings) between the principal component and the four variables were -.701 with health, .874 with happiness, .860 with satisfaction and .481 with participation. We then calculated the extracted component to use it as a dependent variable.

**Fig 1:**
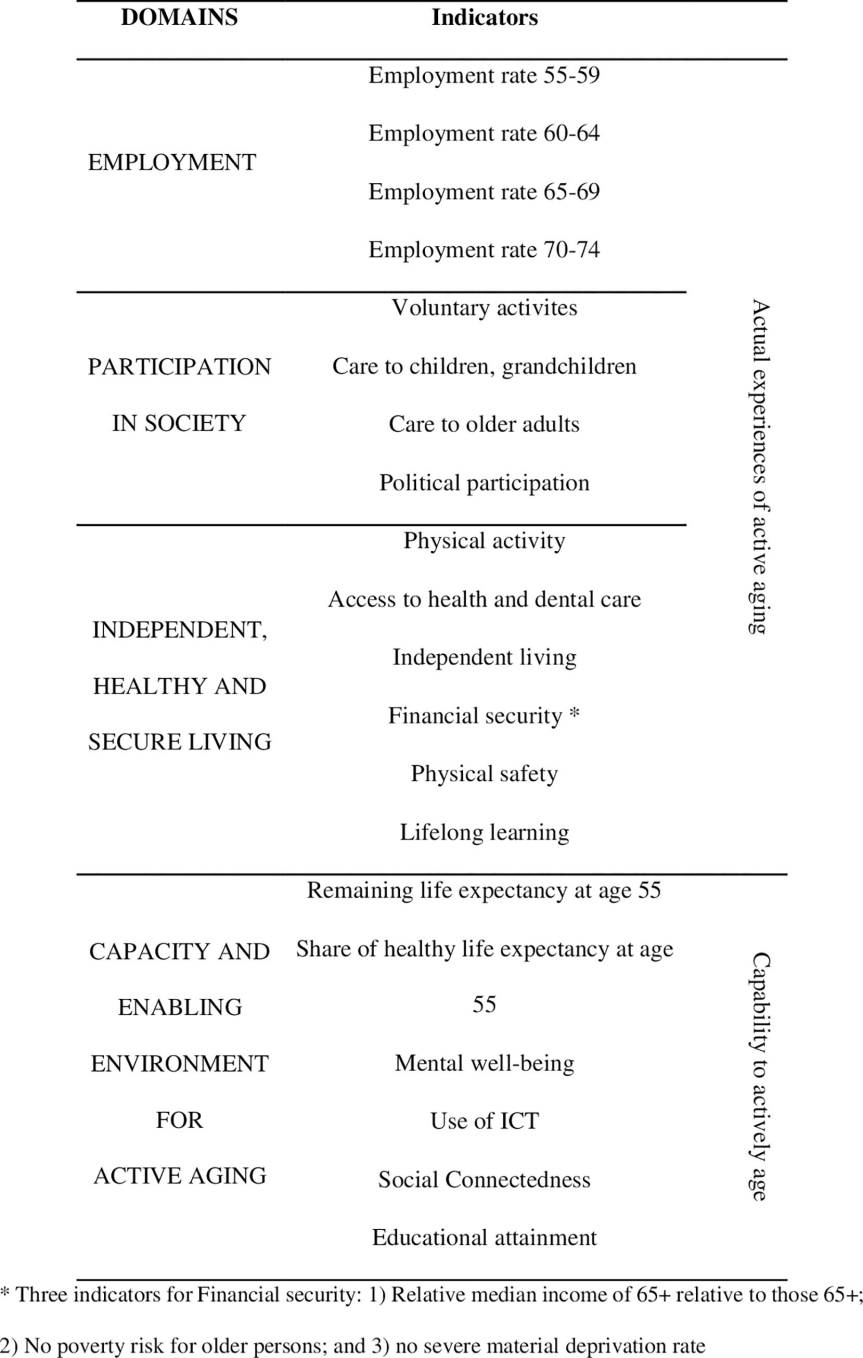
Domains and indicators of Active Aging aggregated index (modified from Zaidi et al. "[48]).

In addition, since the hypothesis mentioned not only interindividual measures, but also population indicators, a population measure was considered: the Active Aging Index (AAI). The AAI was developed under the auspices of the UNECE European Centre for Social Welfare Policy and Research by Zaidi et al. [47]. The AAI provides population indicators composed of four domains operationalized by means of aggregate data from each domain referred to specific countries around the world (Fig 1): 1) Employment 2) Social participation; 3) Independent, healthy and secure living, and 4) Capacity and enabling environment for active aging. Updated country data exist both for the Total AAI and its four components. In summary, we have at our disposal individual data on active aging from the ESS as well as country population data from the European region of the AAI.

### Statistical analysis

With respect to the stereotypical cultural universality hypothesis, a paired *t* test (with Cohen’s *d* effect size) in each country was done to compare population means between the variables warmth (friendly) and competence ("tell me how likely it is that most people in [country] view those over 70s. . . as friendly and. . . as competent”). For a more finely grained understanding of the surveyed countries, we examined the percentages in the cultural stereotype groups and calculated the differences between observed and expected frequencies to show which countries stood out in each of the cultural stereotype groups.

To explicitly test our second hypothesis, multilevel analysis was carried out where level-1 units were individuals (*N* = 7,249 people ≥ 70) and level-2 units were countries (*n* = 29 countries, individuals were nested within countries). The dependent variable was Active Aging, calculated from the ESS questionnaire (see Measurements section above). It is important to note that Active Aging was in a standardized metric (mean = 0 and standard deviation = 1) as it was calculated as factorial scores (see previous section). This facilitates the interpretation of regression coefficients by expressing the predicted change in active aging as a standard deviation metric for each unit that the predictor changes. Thus, Level-1 or individual predictors are the following: (1) stereotype negative vision (1-coded if an individual belongs to this group, 0 otherwise); (2) paternalistic negative vision (again, 1-coded for individuals pertaining to this group, 0 otherwise), then taking the positive vision group as the reference group with which to interpret these dummy variables; (3) Direct prejudice (0–10 scale), (4) perceived discrimination (0–4 scale); and finally, two demographics, (5) female (1-coded for female, 0 for male), and (6) age. All level-1 predictors were group-mean centred [46]. Level-2 or country predictors were the same as mentioned previously (i.e., the individual predictors) but aggregated for each country (averaging by country for people equal to or over 70 years). This makes it possible to find contextual or country effects beyond and above individual effects (at the same time avoiding smushed, or convergent, effects). Thus, 13 fixed effects were tested (one for each predicting variable plus the intercept model). The multilevel model contains two random effects: the residual level-1 variance and the intercept-country variance (in the result section we tested other structures for random variances without improving model fit). The intraclass correlation calculated from the null model was *ICC* = .32 justifying the multilevel framework as there is a large amount of active aging dependency between level-1 units (i.e., individuals) within level-2 units (i.e., countries). The parameters of the model were estimated by restricted maximum likelihood method and the software used was SPSS v20.

In relation to model assumptions, normality of level-1 residuals was checked with descriptive statistics, a box-plot, histogram, and K-S test. Asymmetry was near 0 (*As* = -0.398), as was kurtosis (*K* = 0.360). K-S was significant, but it is very anticonservative with large samples (*K-S* = 0.37, *p* < 0.001), and the histogram and box plot did not show non-normality problems. Level-2 residuals also showed a reasonable fit to normality (*As* = -0.384, *K* = -0.667, *K-S* = 0.087, *p* = 0.345). A pseudo R-square statistic was used as a goodness of fit measure of quality [48].

Finally, with respect to the second hypothesis, we tested the extent to which the aggregated (country) analysis was supported by the population *Active Aging Index* (AAI; [48]) and then established convergent validity of the previous (multilevel) analysis. Pearson correlations were calculated between aggregated (country-level) predictors and the distinct and different subdomains of population AAI.

## Results

### Hypothesis 1: Stereotypical cultural universality hypothesis

Table 2 shows country means for cultural stereotype variables, *t* test statistic under the null hypothesis that population mean difference equals 0, and effect size (Cohen’s *d*). As can be observed, almost all countries showed higher mean in warmth (friendly) than in competence cultural stereotype. Exceptions are Romania and Russia yielding non-significant differences, and Latvia where a higher view of warmth than competence regarding adults over 70s is not held. However, in 26 out of 29 countries, the cultural stereotype of competence being lower than warmth is supported. The average effect size (*d*) was moderate: 0.467 (warmth about half a standard deviation higher than competence).

**Table 2:** Cultural Stereotypes (Friendly and Competent) and paired *t* test statistic and effect size (*Cohen’s d*).

	Most people view those over 70 as friendly	Most people view those over 70 as competent	*t*	*Effect size (d)*
Belgium	2.86	2.51	16.626***	0.397
Bulgaria	2.91	2.60	13.574***	0.300
Switzerland	2.64	2.49	7.647***	0.181
Cyprus	3.14	2.27	27.442***	0.793
Czech Republic	2.75	2.08	26.954***	0.609
Germany	2.74	2.30	22.444***	0.431
Denmark	3.17	2.52	26.714***	0.680
Estonia	2.87	2.80	3.104**	0.078
Spain	3.11	2.61	23.615***	0.481
Finland	3.03	2.71	17.640***	0.380
France	2.82	2.47	16.965***	0.379
United Kingdom	2.97	2.35	33.257***	0.693
Greece	3.10	2.07	40.241***	0.891
Croatia	2.80	1.92	27.507***	0.739
Hungary	3.07	3.01	2.558*	0.066
Ireland	3.35	2.70	30.322***	0.725
Israel	3.11	2.24	37.175***	0.778
Latvia	2.75	2.81	-2.734**	-0.062
Netherlands	2.89	2.54	16.652***	0.399
Norway	3.18	2.67	23.808***	0.608
Poland	2.97	1.86	39.463***	1.009
Portugal	2.85	2.59	13.142***	0.274
Romania	2.49	2.50	-0.220 ^ns^	-0.005
Russia	2.65	2.69	-1.789 ^ns^	-0.037
Sweden	3.12	2.53	26.613***	0.631
Slovenia	2.98	2.03	32.450***	0.915
Slovakia	2.74	2.10	23.602***	0.565
Turkey	2.97	2.26	26.397***	0.557
Ukraine	2.74	2.64	3.623***	0.087
*Total N* = 54,545	*2*.*91 D*.*T*. *= 0*.*93*	*2*.*45 D*.*T*. *= 1*.*03*		

ns = non-significant

* = p<0.05

** = p<0.01

*** = p<0.001

Focusing on more finely grained and subtle cultural stereotype profiles through European countries, Table 3 shows the percentages by country of the negative, ambivalent and positive vision groups. Percentages in bold are for countries that are over-represented in a given category (*z-*standardized residuals above six when comparing observed vs. expected frequencies) and underlined percentages are for countries with observed frequencies six *z-*standardized residuals below the expected frequency. Croatia, Czech Republic, and Romania stand out in the negative vision group (there are many individuals within this group) while Ireland, Sweden, Finland and Norway have very few people in the negative view group. Similarly, Greece, Poland, and Slovenia stand out in the ambivalent group with extremely large percentages, while Hungary, Romania and Russia have very low percentages in this group. Finally, Estonia, Finland, and Hungary are over-represented in the positive vision group, while Croatia, Czech Republic, and Poland have very small percentages. Those not mentioned are countries whose percentages are similar to the European profile and do not stand out in any category. It is worth mentioning that we conducted a k-means and hierarchical cluster analysis to try to find a correspondence between geographical typologies of countries and statistical cultural stereotype profiles. Neither k-means nor hierarchical cluster analysis found country groups that make sense geographically.

**Table 3:** Multilevel model with ESS active aging as dependent variable, *individual* (stereotypical, and demographics) and aggregated (*country*) fixed effects and random effects (model for the variances).

*FIXED EFFECS*	Est.	*S*.*E*.	*t*	*p*	95% Confidence Interval	
					L	U
**Individual variables N = 7.249**						
Intersección	-2.616	11.588	-0.226	.823	-26.646	21.415
Negative vision	-0.116	0.025	-4.648	< .001	-0.165	-0.067
Paternalistic vision	-0.074	0.024	-3.127	.002	-0.120	-0.027
RRDirect prejudice	0.049	0.005	9.049	< .001	0.039	0.060
Perceived discrimination	-0.154	0.010	-16.067	< .001	-0.172	-0.135
Female	-0.009	0.002	-5.311	< .001	-0.013	-0.006
Age	-0.165	0.019	-8.682	< .001	-0.202	-0.127
Aggregated variables n = 29						
Negative vision	-3.043	1.337	-2.275	.033	-5.816	-0.269
Paternalistic vision	0.745	0.812	0.917	.369	-0.940	2.429
Direct prejudice	-0.220	0.209	-1.053	.304	-0.652	0.213
Perceived discrimination	-0.735	0.396	-1.856	.077	-1.557	0.086
Female	0.075	0.137	0.548	.589	-0.208	0.358
Age	-0.893	0.992	-0.900	.378	-2.951	1.165
*RANDOM EFFECTS*						
Residual variance (σ^2^_e_)	0.611	0.010	60.06	< .001	0.592	0.632
Random intercept variance (τ^2^_U0_)	0.127	0.039	3.245	.001	0.069	0.232

Est. = estimation regression coefficient (non-standardized), *S*.*E*. = Standard error, *t* = Wald *t* test, *L* = Lower endpoint and *U* = Upper endpoint.

We carried out the multilevel analysis considering negative vision group as the reference group to test what happened with positive group: as expected, the individual positive vision effect was significantly different from zero: Est. = .102, *p* < .001 and the between-country fixed effect for positive group was significantly different from zero (Est. = 2.30, *p* = .04).

Finally, since European Union countries are usually classified into four regions (Northern, Central, Southern and Eastern, also the four Non-EU countries) we tried to test this geographical aggregate through several statistical procedures based on our stereotypes classification; none of the analyses we performed yielded support for any kind for regional grouping.

In conclusion we can confirm our first hypothesis regarding the universality of the SCM assumption about ambivalent prejudice through individuals and countries as well as the development of a new classification showing a diversity of positive, negative and ambivalent stereotypes of adults over 70s. The following section tests our second hypothesis, that is, the impact of our set of stereotypical concepts on active aging.

### Hypothesis 2: Predicting active aging from individuals and population (countries) levels

In order to test whether and to what extent stereotypes and related concepts predict active aging, two types of analysis where performed: correlational and multilevel, as well as an analysis of convergent validity regarding population and inter-individual active aging measurement.

#### Multilevel model

Taking active aging as dependent variable, the intraclass correlation calculated from the null model (only intercept as fixed-effect and level-1 and level-2 intercept variances as random effects) was *ICC* = .32. Thus, 32% of the active aging variance was attributable to mean differences between countries, and approximately 68% of active aging individual differences occur within countries (around the mean countries). There is a substantive dependence between individuals from the same country. First of all, the moment-product correlations between the stereotypical conditions (positive, ambivalent and negative), direct prejudice and perceived discrimination, although significant (with a high N), are small, concluding that there is no collinearity between predictors.

Table 3 shows the fixed effects (at both individual and country levels) of our stereotypical conditions, that is, negative and paternalistic views (positive group was the reference group), direct prejudice and perceived discrimination as well as demographic conditions such as gender and age.

The individual fixed effects were all significantly different from zero (*p* < .01). They express the expected change in individual active aging within each country for a unit increase in each individual predicting variable. For example, a unit increase in perceived discrimination is associated with a decrease of 0.154 units in active aging, adjusting for the other predicting variables. The negative vision coefficient, -0.116, can be interpreted as the difference in active aging between two individuals (from any country), one belonging to the positive cultural stereotype group and the other to the negative cultural stereotype group (0.116 greater active aging in favour of the individual belonging to the positive vision group). In addition, the significant paternalistic vision coefficient (-0.074) expresses the difference between individuals belonging to paternalistic vs. positive cultural stereotypes groups. As expected, direct prejudice (which expresses positive feelings about older adults) is positively related to active aging, and perceived prejudice is negatively related. Also, age is negatively related with active aging (-0.009 fewer units in active aging with each increased year) and females have less average active aging than males (a difference of 0.165 units). With respect to between-country fixed effects, the negative vision coefficient was the only significant one (*p* = .033); this is important because it supports a country effect of the cultural negative vision on active aging over and above individual effects (that is, countries which differ in negative vision proportions predict different active aging country means). However, non-significant between-country effects were found for the other predicting variables (i.e., no matter how much two countries differ in perceived discrimination averages, or direct prejudice averages, they do not predict significant differences in active aging between these two countries). The final residual variances (level-1 residual variance, σ^2^_e_ and level-2 random intercept variance, τ^2^_U0_) were still significant.

#### Population and inter-individual Active Ageing measures concordance

Since our hypothesis 2 should be tested at individual and population level, it is necessary to obtain the convergent validity from our two measures (ESS Active Aging and population Active Aging Index); thus, Pearson correlations are shown in Table 4.

**Table 4:** Pearson correlations between individual (aggregate/means) measures of Active Aging reported by over 70 individuals (AA-ESS), country proportion of negative (NV), ambivalent (AV) and positive vision (PV), direct prejudice (DP) and perceived (PD) discrimination and the population AAI (and its domains).

	AA-ESS	NV	AV	PV	DP	PD
Active Aging Index (AAI)	.796**	-.515**	-.005	.416*	.036	.226
AA-Employment	.414*	-.277	-.242	.376*	.177	.071
AA-Participation in society	.737**	-.443*	.077	.298^t^	-.177	.347
AA-Independent living	.832**	-.489**	.242	.234	-.101	.338
AA-Capacity for active aging	.835**	-.533**	.139	.334^t^	-.036	.218

*t* = p < .10

* = *p* < .05

** = *p* < .01

Firstly, it is extremely important to note the existence of high correlations between our AA-ESS (which serves as a proxy of population AAI) and the AAI itself. Except with AA-Employment (not used in AA-ESS proxy), AA-ESS correlates more than .700 with AAI-Indices. With overall AAI, the correlation was .796. This result validates that our measures share a high percentage of variance.

Secondly, only the significant correlations between our level-2 predictors and AAI were associated with negative and positive vision. Thus, countries with a higher proportion of people with a negative view of people older than 70 have lower AAI than those countries with a smaller proportion of people with such a view. In sum, the negative view shows the highest (negative) associations with active aging, and no significant associations between active aging and the ambivalent view, direct prejudice and perceived discrimination were found.

## Discussion

It should be emphasized that when verifying hypotheses at individual and population levels in which psychosocial constructs, such as stereotypes, are examined, both descriptively and as predictors of active aging (which contains both objective as well as self-reported or subjective conditions), multiple measures, multiple samples, and multiple analyses should be used, or, in other terms, methodological multiplism is required [45].

Thus, in this study a multiplist approach is used, mainly in order to examine individual self-report measures collected in the *European Social Survey* (ESS-round 4, 2008) in 29 European countries related to health, satisfaction and happiness and social participation, that is, active aging as defined by the WHO [38] as well as objective population indexes from the Active Ageing Index (and its domains, see [48]), the SCM [5, 7, 8, 14] cultural stereotypes towards over 70-year-olds, and two other derivate stereotypical variables such as direct prejudice, and perceived discrimination of over- 70s. We checked for hypothetical associations with individual components of active aging, also in people over 70 (self-perception of health, well-being, happiness, participation) together with their expected associations with population measures yielded by the AAI and its 4 domains by 29 countries participating in the ESS.

### Generalizability of the SCM old-age cultural stereotype: "More warmth than competence"

Regarding our first hypotheses, the use of the two attributes "friendly" and "competent" as stereotypical cultural reagents in our data source (ESS-2008), which asks: "How likely is it that most people, in your country, view those in their 70s. . . as friendly and. . . as competent", has proven to be significant, showing a stereotypical cultural view of the elderly as people who are more friendly than competent in 26 countries out of 29 (excluding Russia, Romania and Latvia). This stereotypical view appears predominant in the vast majority of European countries covered by the ESS, despite the assessment being reduced operationally to the two aforementioned items. Finally, in terms of individuals, 41.9% (*N* = 22,860) scored higher in the warmth variable (“friendly”) than in competence; 46.0% (*N* = 25,108) scored equal in the two variables; only 12.1% (*N* = 6,577) held a view of the over-70s as “more competent than warmth”.

As pointed out in the SCM, "elderly people face a more complex brand of prejudice that comprises both negative and subjectively positive beliefs, feelings, and behaviours” [5, 7, 8]. This implies an ambivalent cultural vision, and thus supports the universality of this "ambivalent" cultural attitude regarding older adults, called "paternalist prejudice" by the SCM, and as found in other studies in the USA [1, 5, 7,51], European countries (Belgium, France, Germany, The Netherlands, Portugal, Spain, and the UK), and three East Asian countries (Hong Kong, Japan, and South Korea) (10). Finally, this was also found by our own research group in different samples from Spain and Mexico in several samples of general population as well as in caregivers in older adults [1, 12, 37, 49, 50].

A step forward has been made classifying individuals and countries through a positive, ambivalent and negative cultural view about people over 70. At this point, we should emphasize the empirical support for the assumption that negative self-stereotypes (in comparison with positive) are those linked negatively with health (e.g.: [4, 41]). Our SCM ambivalent view–generalized across people and countries–resides in two positive human characteristics, one affective (warmth) and the other intellectual (competence). In the words of Cuddy and Fiske [22], the aging stereotype of older persons in the comparison of these characteristics (W>C) as “*Doddering but Dear*”, is highly negative. Based on the characteristics of the ESS W and C measures, and with the objective of arriving at a positive/negative view which also included the ambivalent dimension, it was decided to classify countries, following a metric already described in the Method section, into three groups: negative, positive and ambivalent.

Our nuanced classification by countries shows that almost half (46.62%) the European countries have a positive cultural view towards over 70-year-old adults, almost one third (30.50%) reported an ambivalent view and almost a quarter (22.88%) held a negative stereotypical view.

This stereotypical cultural view can be evaluated through a personal focus on the older adult’s personal appraisal. As already stated, the ESS includes an item evaluating the over-70s (called direct prejudice by Vauclaire et al. [43]) through the question: "Tell me overall, how negative or positive you feel towards people over 70?" (scale range: 0–10). This personal appraisal yields a mean score of 7.58 (SD = 1.81) on a scale of 0–10, providing a positive assessment of people over 70, well above the arithmetic mean of the scale used (negative/positive). If we add to this the low correlation between the personal assessment and the cultural view of the "friendly" and "competent" dimensions and their difference (r = .251, r = .171 and r = .053, respectively), we may conclude that both constructs (cultural stereotype and personal valuation) share only 6% of the common variance. As most authors agree (for a review, see [51]), the cultural (perceptual/cognitive) view can be considered conceptually different from a personal affective appraisal. This is not only the case because they allude to different psychological functions, but also because the cultural view seems to contain an automatic component less prone to social desirability or any other response tendency of self- or first-person reports. This is confirmed when defining a negative cultural view operationally and contrasting it with a negative personal assessment–it is always the negative cultural view that explains a greater proportion of variance in all variables analysed, both individual and population, which comprise active aging.

Despite this, the use of positive reagents does not prevent the appearance of a negative stereotypical view when a certain social group is attributed with high warmth and low competence for two different reasons: 1) in our society, competence is a highly valued social construct in the social world and the world of work and is highly influential in important decisions such as employment. However, in our data, it should also be noted that only 7% of the 54,545 individuals surveyed are attributed as more competent than friendly to older adults, and 2) both attributes are measurable, and comparing them depends not only on the significance of the differences between them, but also on the basal value of both, so that, when attributed to a limited extent, can become negative ("not very competent and not very friendly"). For these key reasons, we have established a classification (see Table 1) in which (after eliminating the infrequent construct of high competence), we have compared (via individuals and via countries) to what extent a negative, ambivalent view (significantly more friendly than competent) or a positive view is associated with a series of variables related to active aging.

In sum, based on the European Social Survey, the higher-warmth-than-competence cultural view about older people postulated by the Stereotype Content Model (SCM) has been found in most European Countries (26/29). However, when a more sophisticated older adult cultural view classification (positive, ambivalent, and negative), based on friendliness and competence scores, is used, only 28.4% of Europeans showed an ambivalent view about older adults, while 41.7% reported a positive view and 21.3% reported a negative view. It is remarkable that only a 7% see older adults as competent (i.e., more competent than friendly).

### To what extent are cultural stereotypes, and their theoretical consequences, prejudice and discrimination a risk to active aging?

Our results with both individual and population data highlight the fact that a negative stereotypical cultural view in a country is significantly and negatively associated with different characteristics reported by over-70s regarding their health, satisfaction and happiness, and social network–all components of active aging, as defined by the WHO [35]). These results are also supported by our multilevel analyses but with considerable differences at individual and contextual impact levels. Thus, at individual levels, age and gender showed broadly significant negative differences by sex–women and age as well as all our negative and ambivalent stereotypical variable views–as population conditions, direct prejudice, and perceived discrimination significantly predicted Active Ageing reported in the ESS. Nevertheless, only negative cultural stereotypes significantly predicted ESS active aging.

From a methodological point of view, perhaps more importantly, our indexes of active aging self-reported through the ESS and AAI correlate at around .80. Similarly, data yielded by our contextual analysis showed that a negative cultural view reported in a country is negatively and significantly associated with the global Index of Active Aging (AAI). An examination of the stereotype indexes by partial population components showed that they all present significant negative associations. It can be concluded that a negative view of older adults is associated with lower participation, independent life and health and an enabling environment for active aging. The only non-significant association corresponded to Employment, which is operationalized (see Fig 1) by people in work (by age groups) from 55 to 74 years of age; this is more dependent on the labour legislation of each country than on the active aging of the population itself.

In summary, negative stereotypes are highly associated with low population Active Aging Index. These data are supported also by our multilevel analysis, only negative cultural views are associated significantly (p < .01) with both individual and population active aging. These results are all in agreement with authors such as Swift, Abrams, Lamont and Drury [40], who postulate that negative stereotypes pose a risk for active aging. Data from those over 70 in the ESS confirm this in relation to their health, satisfaction, happiness and participation; furthermore, these are all components of active aging and other related concepts (for a review see [52]). Nevertheless, although direct prejudice and perceived discrimination significantly predict inter-individual differences in active aging reported in the ESS by adults over 70, this prediction is not significantly supported at contextual nor population levels when an aggregate indicator such as the AAI is used.

These results are probably due, to some extent, to differential individual and context variances to be explained in multilevel analysis, and to the different sample sizes in the two levels (where only 29 countries but more than 7,000 individuals or level-1 units participated in the multilevel model). Nevertheless, the remarkable point here is the relative importance of contextual (aggregated) effects tested in our models: we found that, at the country level, cultural stereotypes are more powerful in explaining active aging than more directly assessed constructs such as direct prejudice or perceived discrimination.

Conversely, this study shows that a positive cultural outlook in certain contexts appears to be associated with successful or active aging promotion (for a review see [53]). This has been demonstrated in previous experimental studies which highlight that programs for the promotion of active aging with modules to reduce negative stereotypes on aging obtain better results in the target variables (health, positive affect, etc.) [39, 54, 55]. Thus, in evaluation studies of promotion programs for older adults, performed under quasi-experimental designs (pre/post-test, with no-equivalent control group), carried out in four countries (Spain, Cuba, Mexico and Chile; see [39]), participants positively modified both their stereotypical cultural views about older adults as well as their self-perception of aging (see also: [27]).

Nevertheless, we may ask which mechanism is working in the dense and complex network of cultural stereotypes in the interchange of social health welfare systems and the older adults' individual behaviours, mainly because, after a short-term intervention (about three months), the change expressed in the post-test about cultural stereotypes refers only to an individual meta-perception change and cannot be explained by the theory proposed by Levy [23] regarding the *embodiment model* described above. Nevertheless, the changes in self-perception of aging could be better understood given the main emphasis on active aging lifestyles and the probability of aging well in the development of all countries involved.

The present work drawing on SCM advances a classification of cultural views, predominant in different nations, based on competence and warmth attributed to people over 70 which appears to be very useful in revealing the association with health, satisfaction, happiness, social relationships, all of them outputs of active aging [36]. Moreover, at the societal level, negative cultural views about over-70s are negatively associated with the Active Aging Index-AAI (and most of its components). It is interesting to underline how competence vs. warmth views regarding over-70s predict population and personal conditions. On the one hand, the SCM competence and warmth dimensions are formulated positively and refer to the collective view of the group, thereby hiding the latent evaluative dimension and are thus perhaps less subject to social desirability than direct personal evaluation. On the other hand, by classifying nations in terms of the positive, ambivalent, and negative views of older people, we were able to make the evaluation prevalent in each society more explicit, which may be helpful for furthering knowledge of the underlying mechanisms.

This study has made novel contributions by analysing data from the European Social Survey (ESS- 2008) in 29 European countries using both individual indicators from older adults and population aggregated indicators by country in a multilevel model. First of all, the operationalization of the cultural stereotype has allowed us to establish a typology of countries, ascribing values to them according to their negative, ambivalent, and positive attitudes. Our results show that the cultural view about older adults as high warmth and low competence is widespread in most European countries, and that a negative cultural view of older adults is significantly and negatively associated with different characteristics reported by over-70s regarding their health, satisfaction and happiness, and social network, all conditions of active aging, leading to the conclusion that it does indeed represent a threat to satisfactory, healthy and active ageing.

**Fig 2:**
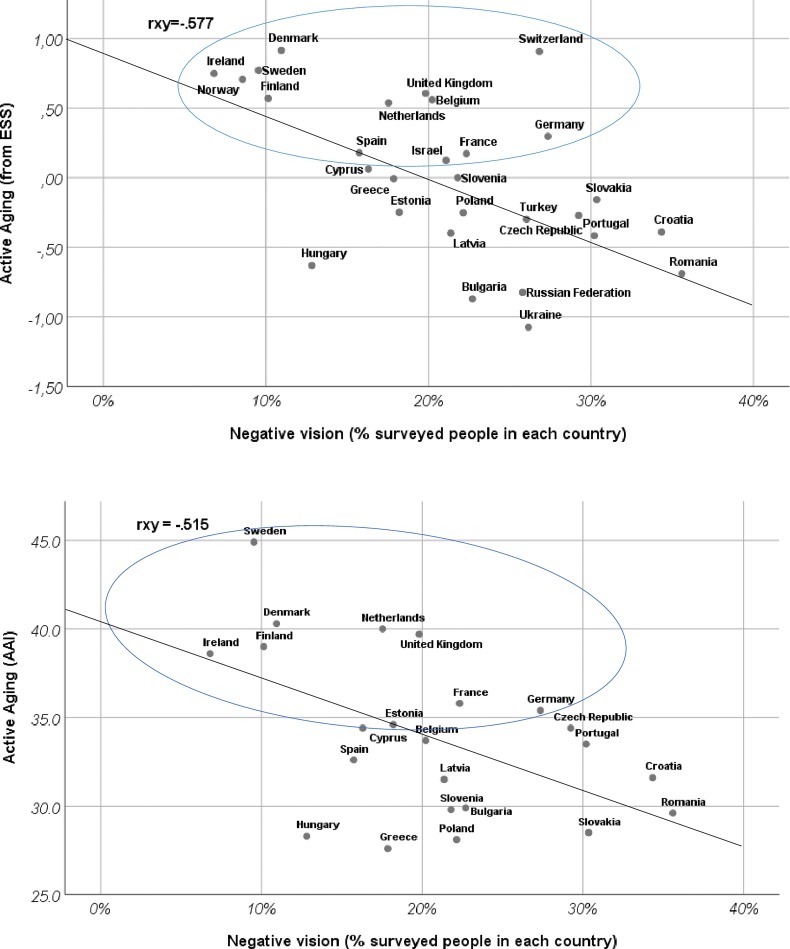
Cultural negative views and Active Aging (individual reported- ESS and population- AAI) by countries of the European Region.

Although we did not obtain a regional classification based on our classification (negative, ambivalent and positive), this classification allows us to draw two conclusions: 1) Although following the SCM metric regarding the balance among warmth and competence most (26/29) European Countries support the SCM universality of an ambivalent view regarding older adults, when we tried to enlarge this metric and polarized individual stereotypical responses at the country level already described, an ambivalent view is less prevalent (28%) than a positive view (47%). 2) When we examined the extent to which negative, ambivalent or positive views of over-70s predict or are associated with active aging, both at individual or population levels, the negative view was the best negative predictor of active aging. Fig 2 shows the relationships between both Active Aging at an individual level (AA-ESS) and at a population level (Active Aging Index-AAI), which can be considered a synthesis of our work.

As we can observe, both graphs in Fig 2 show very similar patterns, a visual reinforcement that Active Aging-ESS and Active Aging Index-AAI are two very related measures, and that the active aging proxy calculated from ESS individuals is definitely a valid measure of the population index (i.e., Active Aging Index-AAI). Also, the presence of a geographical distribution of the countries in the scatter plot is remarkable. While we did not find a natural correspondence between a geographical distribution of countries and the clusters based on cultural stereotypes percentages, when active aging and cultural stereotypes are plotted jointly, this correspondence is more evident. In the upper left we find the Scandinavian countries (e.g., Denmark, Norway, Finland), in the middle are Central European and Southern countries (e.g., Germany, France, Spain, Greece; it must be remembered that Italy is not in the ESS) and predominantly East European countries such as Russian and Romania are found in the lower right. Of course, the clustering is not perfect, but the correspondence with the geographical situation and where the countries are placed in the scatter plot is notable.

The main conclusions of this study are the following: 1) Although the universality of the cultural stereotype supported by the SCM [5] about older adults as more “friendly” than “competent” has been positively tested, its predictive power or negative association with health and active aging (assessed both by population and inter-individual data) is not confirmed; 2) Nevertheless, as has been claimed by Palmer [40] or Levy [56], among others, the predictive power of negative (and positive) stereotypes of health and active aging is tested both through multilevel inter-individual and aggregate analyses; 3) Finally, the hypothesis deriving from ageism [2] and from Swift et al. [40] regarding a strong link between cultural stereotypes, direct prejudice or perceived discrimination was not tested; from our analyses, only “negative” cultural stereotypes were a threat for active/healthy aging. To close, it should be said that this conclusion needs to be tested, overcoming methodological flaws in the assessment of those psychosocial constructs. Although this study has various strengths, several limitations should be acknowledged. First, ageing stereotyping was measured explicitly using two items rather than using the SCM-questionnaire. Secondly, the effects of stereotyping on active aging seem clear through individual (stereotypical, and demographics) variables rather than through aggregated (country), which might be due to social desirability and the measures used. Finally, this is a cross-sectional study, but we are planning future longitudinal and cohort research continuing with this fascinating issue.
